# Advances in assessment systems and non-pharmacological intervention strategies for cancer-related fatigue following transarterial chemoembolization in hepatocellular carcinoma: a narrative review

**DOI:** 10.1007/s00520-026-11380-9

**Published:** 2026-10-23

**Authors:** Shiying Yang, Xuelei Yao, Yali Ren, Teng Ding, Jiamin Gao, Lin Shentu

**Affiliations:** 1 Department of Nursing, Fudan University Shanghai Cancer Center, Department of Oncology, Shanghai Medical College, Fudan University, Shanghai, 200032 China; 2 Department of Nursing, Changhai Hospital, First Affiliated Hospital of Naval Medical University, Shanghai, 200433 China; 3 Present Address: Shanghai Songjiang Sijing Hospital, Shanghai, 201601 China

**Keywords:** Cancer-related fatigue, Hepatocellular carcinoma, Transarterial chemoembolization, Assessment tools, Non-pharmacological intervention, Nurse-led management

## Abstract

**Purpose:**

Transarterial chemoembolization (TACE) is a primary treatment for intermediate to advanced hepatocellular carcinoma (HCC); however, cancer-related fatigue (CRF) is a prevalent and distressing symptom following TACE that significantly impairs patients' recovery and health-related quality of life. This review aimed to systematically summarize the current evidence on CRF screening, assessment, and non-pharmacological interventions specifically tailored to the post-TACE HCC population, and to propose an integrated, nurse-led clinical management framework.

**Methods:**

A narrative review was conducted by systematically searching PubMed, Web of Science, and CNKI for literature published up to April 2026. The search encompassed peer-reviewed original research articles (including randomized controlled trials, cohort studies, cross-sectional studies, and meta-analyses), clinical practice guidelines, and expert consensus reports. The level of evidence for each intervention category was evaluated using the Oxford Centre for Evidence-Based Medicine (OCEBM) grading system. The literature search focused on CRF epidemiology, pathophysiology, validated assessment tools, non-pharmacological interventions (including exercise, psychosocial support, nutritional management, and traditional Chinese medicine therapies), and nursing management models in HCC patients following TACE.

**Results:**

CRF following TACE is a multifactorial symptom driven by treatment-induced inflammation, hepatic dysfunction, and psychological distress. Validated screening tools, including the Brief Fatigue Inventory (BFI), Cancer Fatigue Scale (CFS), and EORTC QLQ-C30 fatigue subscale, are recommended for routine use, though to the best of our knowledge, no CRF assessment tool developed specifically for the post-TACE HCC population has been validated to date. A structured screening pathway encompassing baseline assessment, perioperative monitoring, and long-term follow-up was identified as essential for early detection. Among non-pharmacological interventions, individualized exercise prescriptions (aerobic and resistance training) have been most consistently supported across randomised trials and meta-analyses for alleviating CRF, followed by cognitive behavioral therapy, mindfulness-based stress reduction, and nutritional support. Traditional Chinese medicine approaches, including acupoint massage, auricular acupressure, and moxibustion, showed preliminary potential but require further high-quality validation. A nurse-led multidisciplinary care model integrating assessment, individualized intervention planning, and continuous coordination has been proposed as a promising operational framework for CRF management, endorsed by international CRF practice frameworks.

**Conclusions:**

Effective management of CRF after TACE requires a comprehensive, evidence-based approach spanning standardized screening, multimodal non-pharmacological interventions, and a nurse-coordinated multidisciplinary care model. Integrating routine CRF assessment into standard TACE clinical pathways and developing TACE-specific assessment tools are priorities for future research and clinical practice.

## Introduction

Hepatocellular carcinoma (HCC) is a highly prevalent malignancy worldwide with diverse treatment modalities, among which transarterial chemoembolization (TACE) serves as the primary palliative treatment for intermediate to advanced HCC, demonstrating significant efficacy in controlling tumor progression [1]. Multiple studies have confirmed that TACE plays a central role in the management of unresectable HCC, achieving tumor control through local chemotherapy and embolization of tumor-supplying arteries [2]. However, TACE therapy itself and its associated post-embolization syndrome and hepatic functional changes are important precipitating factors for cancer-related fatigue (CRF) in patients [3]. CRF is a distressing, persistent, subjective sense of exhaustion related to cancer or its treatment, disproportionate to recent activity, and not fully relieved by rest. This fatigue is highly prevalent among HCC patients undergoing TACE but is often overlooked by healthcare professionals or attributed to the disease itself, lacking systematic assessment and management [4].

TACE is frequently combined with other therapeutic modalities to enhance efficacy, such as tyrosine kinase inhibitors (e.g., sorafenib, lenvatinib) or immune checkpoint inhibitors [5]. However, while combination therapy improves objective response rate (ORR), disease control rate (DCR), and overall survival (OS), it also brings a higher incidence of adverse events, among which fatigue is a common adverse effect [6, 7]. For instance, a meta-analysis comparing sorafenib combined with TACE versus TACE alone demonstrated a significantly higher incidence of fatigue in the combination therapy group [6]. Similarly, in randomized controlled studies comparing conventional TACE (cTACE) with drug-eluting bead TACE (DEB-TACE), the incidence of fatigue was significantly higher in the cTACE group than in the DEB-TACE group [8]. These findings suggest that fatigue is an important negative factor affecting patients' quality of life, regardless of whether TACE is administered as monotherapy or in combination.

Among the HCC population undergoing TACE, the development of CRF is associated with multiple interrelated factors. A systematic review has indicated that health-related quality of life (HRQoL) in post-TACE patients is influenced by a complex interplay of demographic factors, clinical indicators, psychological factors, social support, and physical symptoms (e.g., fatigue, pain, and loss of appetite) [4]. As a core physical symptom, the severity of fatigue is positively correlated with psychological distress (e.g., depression and anxiety), collectively contributing to the decline in patients' quality of life [9]. Furthermore, patients' self-efficacy and level of social support are closely associated with the degree of CRF, with patients exhibiting lower self-efficacy and inadequate social support tending to experience more severe fatigue [10, 11]. Clinical characteristics such as prolonged disease course, poor Child–Pugh classification, and receipt of adjuvant chemotherapy have also been confirmed as independent risk factors for CRF [12]. Notably, post-TACE fatigue is not an isolated symptom; it frequently co-occurs with post-embolization syndrome (e.g., fever, nausea, vomiting, and abdominal pain) and abnormalities in liver function parameters (e.g., elevated transaminases and bilirubin), and these treatment-related responses further exacerbate patients' physiological and psychological burden, creating a vicious cycle [8, 13].

.In recent years, with the development of the "patient-centered" healthcare model, increasing attention has been directed toward the quality of life of post-TACE patients, and CRF management has become one of the core components of supportive cancer care. Despite its high prevalence and profound impact, CRF remains frequently underrecognized and undertreated in clinical practice [14, 15].

Therefore, a systematic review of CRF assessment and intervention strategies is crucial for improving the overall prognosis of HCC patients undergoing TACE. Based on existing research evidence, this article systematically reviews the current status and progress in this field from three dimensions—screening and assessment, non-pharmacological interventions, and nursing management—with the aim of providing a reference for developing individualized fatigue management protocols in clinical practice. Existing interventional studies have demonstrated that non-pharmacological approaches such as exercise training (e.g., aerobic exercise and walking exercise), psychological interventions (e.g., solution-focused approach and mindfulness-based stress reduction), and traditional Chinese medicine (e.g., Jianpi Xiaoji Decoction and Jianpi Yangrong Decoction) have shown positive effects in alleviating CRF and improving quality of life in HCC patients [16–21]. These advances have laid a solid foundation for constructing a multidimensional, integrated CRF management strategy.

## Epidemiology and pathophysiological mechanisms of CRF in HCC patients undergoing TACE

### Incidence and influencing factors of Post-TACE CRF

Among patients with HCC, the incidence of cancer-related fatigue (CRF) following transarterial chemoembolization (TACE) is exceedingly high and severely impairs their quality of life. Multiple studies have confirmed that TACE is a key modality for treating intermediate to advanced hepatocellular carcinoma (HCC) [22, 23]. However, this treatment modality itself introduces a series of side effects, among which fatigue is particularly prominent. Although the existing literature does not directly provide specific incidence data, the widespread application of TACE as a first-line recommended therapy for intermediate to advanced HCC implies that post-TACE CRF is a prevalent concern [23]. The factors influencing post-TACE CRF are multidimensional and complex. Regarding treatment-related factors, the efficacy and complications of TACE are closely associated with tumor characteristics and hepatic functional status. For instance, patients with smaller tumor burden (e.g., meeting the up-to-seven criteria) and better hepatic function (Child–Pugh class A) demonstrate better responses to TACE, which may indirectly affect the severity of fatigue [24, 25]. Conversely, factors such as larger tumor diameter, more advanced BCLC staging, and poorer hepatic function (Child–Pugh class B) are associated with worse prognosis, and these factors may also exacerbate patients' fatigue symptoms [26]. Furthermore, TACE treatment itself may induce inflammatory responses; for example, post-procedural infectious complications such as liver abscesses can further deplete patients' physical capacity and aggravate fatigue [27, 28]. Psychosocial factors, such as the stress of facing advanced cancer and repeated treatments, undoubtedly constitute important precipitating factors for CRF; although not directly elaborated in the reviewed literature abstracts, their impact should not be overlooked based on clinical experience.

### Pathophysiological basis of CRF and the specificity of TACE

**Fig. 1:**
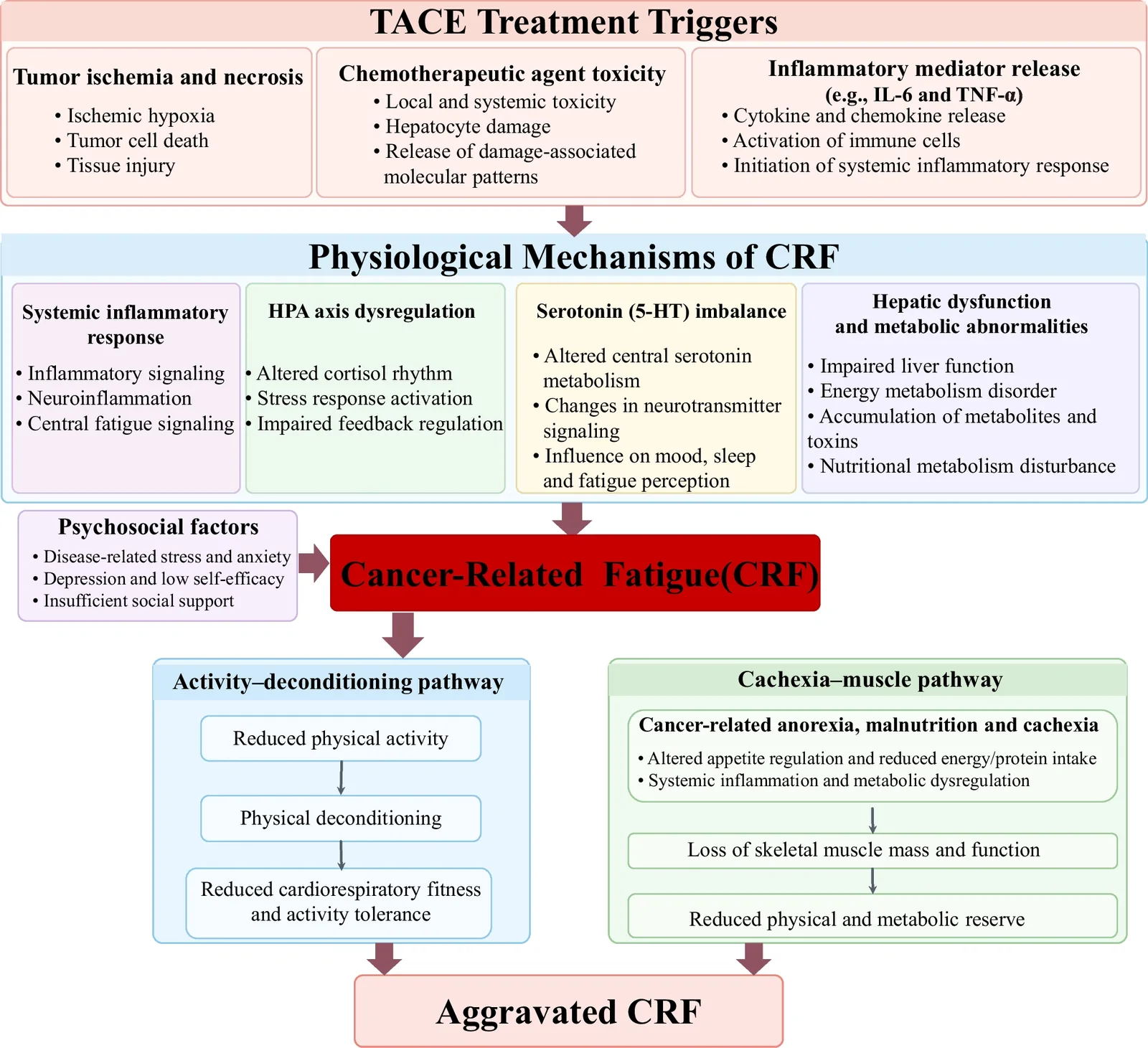
Pathophysiological Mechanisms of CRF After TACE. Abbreviations: TACE, Transarterial Chemoembolization; CRF, Cancer-Related Fatigue; HPA, Hypothalamic–Pituitary–Adrenal; 5-HT, Serotonin; IL-6, Interleukin-6; TNF-alpha, Tumor Necrosis Factor-alpha

The pathophysiological mechanisms of cancer-related fatigue involve multiple pathways, including inflammation and metabolic dysregulation, and TACE therapy may significantly exacerbate these processes through its unique mechanism of action Fig. 1. The core principle of TACE is to embolize tumor-supplying arteries and locally infuse chemotherapeutic agents to induce tumor ischemia and necrosis [2]. This process releases a large quantity of inflammatory mediators and cytokines, triggering a systemic inflammatory response, which is considered one of the central mechanisms underlying CRF. For example, both drug-eluting bead TACE (DEB-TACE) and conventional TACE (cTACE) cause dramatic changes in the local tumor microenvironment [29]. The acute tissue injury and inflammation caused by TACE may disrupt hypothalamic–pituitary–adrenal axis function, affect energy metabolism, and alter neurotransmitter balance (e.g., serotonin), thereby producing persistent fatigue. The specificity of TACE is also reflected in its impact on the liver itself and the portal venous system. Treatment may cause transient hepatic functional impairment, affecting the body's energy metabolism and toxin clearance capacity [30]. More uniquely, for patients with advanced HCC accompanied by portal vein tumor thrombus (PVTT), TACE requires highly refined techniques (e.g., superselective TACE); however, even with meticulous technique, the treatment's impact on portal venous hemodynamics and potential bile duct injury add complex mechanisms for CRF that differ from other cancer treatments [31]. These factors collectively result in post-TACE fatigue that is not only highly prevalent but also has a more complex pathophysiological basis.

## Screening methods and standardized assessment tools for CRF

### Application of generic and disease-specific assessment tools

In clinical practice, for the assessment of cancer-related fatigue following transarterial chemoembolization in hepatocellular carcinoma, multiple validated instruments are used to quantify the severity of fatigue and its impact on patients' lives Table 1. The Brief Fatigue Inventory (BFI), as a concise tool using a 0–10 numerical rating scale, can rapidly capture the severity of patients' fatigue over the past 24 h and its interference with daily activities, facilitating preliminary screening in busy clinical settings [14, 32]. The Cancer Fatigue Scale (CFS) is similarly widely used due to its brevity and favorable psychometric properties; it assesses fatigue across three dimensions—physical, affective, and cognitive—and has demonstrated reliable validity among cancer patients from diverse cultural backgrounds [33]. For research and clinical situations requiring more comprehensive, multidimensional assessment, the European Organisation for Research and Treatment of Cancer Quality of Life Questionnaire-Core 30 Fatigue Subscale (EORTC QLQ-C30-FA) is one of the most commonly used instruments for evaluating health-related quality of life, and its fatigue subscale effectively reflects patients' fatigue symptoms [34]. Additionally, the Multidimensional Fatigue Inventory (MFI-20) was designed to comprehensively assess five dimensions of fatigue: general fatigue, physical fatigue, reduced activity, reduced motivation, and mental fatigue [34]. However, to the best of our knowledge, no CRF assessment tool developed specifically for the post-TACE HCC population has been validated to date [34, 63, 64]. Existing generic instruments were not designed with the hepatic functional impairment and post-embolization syndrome unique to TACE in mind, and the absence of TACE-specific normative data limits their interpretive precision in this population. Existing studies predominantly rely on the aforementioned generic instruments; however, in clinical interpretation of their results, the instrument scores must be integrated with patients' objective clinical indicators for comprehensive analysis, such as hepatic functional reserve (e.g., Child–Pugh classification) and performance status scoring (e.g., ECOG PS) [14]. This integrated assessment facilitates a more accurate understanding of the clinical significance of fatigue and provides a basis for subsequent management.

**Table 1:** CRF assessment tools applicable to Post-TACE HCC patients

Assessment Tool	Full Name	Items/Scoring	Dimensions Assessed	Strengths	Limitations	Applicability to Post-TACE HCC	Completion Time
BFI	Brief Fatigue Inventory	9 items; 0–10 numerical scale	Severity + interference with daily function	Rapid screening; validated across multiple languages and cancer types; strong psychometric properties (α = 0.82–0.97)	Single dimension; does not capture emotional or cognitive aspects of fatigue	Well-suited for rapid perioperative screening and longitudinal monitoring; widely validated in cancer populations	< 5 min
CFS	Cancer Fatigue Scale	15 items; 1–5 Likert scale	Physical, affective, and cognitive	Three-dimensional assessment; good psychometric properties (α = 0.88 [Okuyama 2000]; 0.94 [Robinson 2025]); extensive cross-cultural validation	More items than unidimensional tools; may be burdensome for severely fatigued patients	Suitable for multidimensional assessment; validated in diverse cancer populations	~ 5 min
EORTC QLQ-C30 FA	EORTC QLQ-C30 Fatigue Subscale	3 items; 1–4 Likert scale	Fatigue severity	Integrated with comprehensive HRQoL evaluation; sensitivity 86%, specificity 78%	Only 3 items; limited depth of fatigue characterization	Useful when HRQoL monitoring is needed alongside fatigue screening	< 1 min
MFI-20	Multidimensional Fatigue Inventory-20	20 items; 5-point Likert scale (1–5)	General fatigue, physical fatigue, reduced activity, reduced motivation, mental fatigue	Comprehensive five-dimension profiling; good internal consistency (α = 0.79–0.90 [Westenberger 2022]; ωt = 0.964)	Longer completion time; some cross-loading issues in translated versions	Appropriate for in-depth multidimensional assessment when detailed fatigue profiling is clinically indicated	5–10 min
NRS	Numeric Rating Scale	1 item; 0–10 numerical scale	Overall fatigue severity	Extremely simple; suitable for daily bedside screening; recommended by NCCN for initial screening	Too brief for comprehensive assessment; no multidimensional information	Ideal for rapid triage and serial monitoring in busy clinical settings	< 1 min

Abbreviations: *BFI* Brief Fatigue Inventory, *CFS* Cancer Fatigue Scale, *EORTC QLQ-C30 FA* European Organisation for Research and Treatment of Cancer Quality of Life Questionnaire–Core 30 Fatigue Subscale, *MFI-20* Multidimensional Fatigue Inventory–20, *NRS* Numeric Rating Scale, *HCC* Hepatocellular carcinoma, *TACE* Transarterial chemoembolization, HRQoL Health-related quality of life. Applicability assessments are based on the tool’s validation status in cancer populations, item burden, and relevance to the post-TACE clinical context. Cronbach’s α values represent the range reported in published validation studies. For detailed psychometric properties and literature citations, please refer to Sect. "Application of generic and disease-specific assessment tools" of the main text. Completion time estimates are derived from published literature.

### Appendix: psychometric properties of CRF assessment tools

#### Brief Fatigue Inventory (BFI)

The Brief Fatigue Inventory (BFI), developed by Mendoza et al. [52], is a nine-item instrument designed for the rapid assessment of fatigue severity and its interference with daily function. Across multiple validation studies, the BFI has consistently demonstrated excellent internal consistency (Cronbach's α > 0.90) [37]. Concurrent validity was supported by significant correlations with established measures including the Profile of Mood States fatigue subscale and the SF-36 vitality scale in the original development study [52]. The Chinese version (BFI-C) was subsequently validated by Wang et al. [53] in a sample of 249 cancer patients with multiple diagnoses, yielding Cronbach's α of 0.92 for the severity items and 0.90 for the interference items. Known-groups validity has been demonstrated through significant differences in BFI scores across ECOG performance status categories [52].

#### Cancer Fatigue Scale (CFS)

The Cancer Fatigue Scale (CFS), developed by Okuyama et al. [54], is a 15-item self-rating instrument comprising three subscales: physical, affective, and cognitive fatigue. The original Japanese validation study reported a Cronbach's α of 0.88 for the total scale [54]. Fisher et al. [37], in their systematic review of CRF assessment tools, confirmed the robust psychometric properties of the CFS across diverse cultural settings, with adequate convergent validity established through significant correlations with the Fatigue Visual Analogue Scale, the Fatigue Symptom Inventory, and the Hospital Anxiety and Depression Scale. Discriminant validity was supported by the scale's ability to differentiate patients with different Karnofsky Performance Status scores (> 80% vs. < 80%) and varying levels of psychological distress [37]. More recently, Robinson et al. [55] validated the CFS in a UK population (*N* = 160), reporting a total scale Cronbach's α of 0.94, with subscale α values of 0.92 (physical), 0.87 (affective), and 0.83 (cognitive). Principal component analysis supported the original three-factor structure, accounting for 64% of the total variance. Convergent validity was further confirmed through significant correlations with the EORTC QLQ-C30 fatigue subscale (ρ = 0.78) and the HADS depression subscale (ρ = 0.78).

#### EORTC QLQ-C30 Fatigue Subscale

The three-item fatigue subscale of the EORTC QLQ-C30 is one of the most widely used measures of CRF in clinical trials. According to the systematic review by Fisher et al. [37], the fatigue subscale has shown acceptable internal consistency (Cronbach's α = 0.67–0.84). The subscale demonstrated moderate correlations with functional limitations and the need for assistance in daily activities, supporting its convergent validity. For diagnostic accuracy, the fatigue subscale showed a sensitivity of 86% and a specificity of 78% for identifying clinically significant fatigue [37].

#### Multidimensional Fatigue Inventory-20 (MFI-20)

The Multidimensional Fatigue Inventory (MFI-20), originally developed by Smets et al. [56], is a 20-item instrument measuring five dimensions of fatigue: general fatigue, physical fatigue, reduced activity, reduced motivation, and mental fatigue. The original Dutch validation study supported the five-factor structure with adequate internal consistency for the total scale [56]. The Chinese version was validated by Tian and Hong [57] in patients with cancer, with Cronbach's α exceeding 0.80 for the total scale and 0.70 for each dimension. However, factor structure has remained debated. Westenberger et al. [58], in a large representative German population sample (*N* = 2,509), reported excellent overall reliability (McDonald's ωt = 0.964) with subscale α values ranging from 0.79 (reduced motivation) to 0.90 (physical fatigue), but found that confirmatory factor analysis did not support the original five-factor model (CFI = 0.891, TLI = 0.871, RMSEA = 0.105). They recommended using the total score or the general fatigue subscale as the primary fatigue indicator, and provided age- and sex-stratified normative data for clinical interpretation.

#### Numeric Rating Scale (NRS)

The single-item NRS (0–10) is the simplest screening tool for CRF and is recommended by the NCCN Guidelines for initial fatigue screening at each clinical visit [59]. Because of its single-item nature, Cronbach's α cannot be calculated; however, test–retest reliability has been reported as adequate (intraclass correlation coefficient ≥ 0.70) [37]. Concurrent validity is supported by moderate-to-strong correlations with multidimensional fatigue measures, including the BFI (r ≥ 0.70) [37]. The primary limitation of the NRS is its inability to capture the multidimensional nature of CRF, making it most appropriate for rapid screening rather than comprehensive assessment.

#### Guideline recommendations for assessment tools

 The NCCN Guidelines for Cancer-Related Fatigue (Version 2.2025) recommend routine fatigue screening for all patients with cancer at the initial visit, at regular intervals during and following treatment, and as clinically indicated, using age-appropriate measures, including a 0–10 numerical rating scale (NRS) [59]. The ESMO Clinical Practice Guidelines for cancer-related fatigue [60] similarly emphasize routine screening using validated instruments, recommending unidimensional scales (e.g., NRS, BFI) for rapid screening and multidimensional tools (e.g., MFI-20, EORTC QLQ-C30) for comprehensive assessment. The APTA Oncology Section clinical practice guideline, based on a systematic review of psychometric evidence, rated the BFI and CFS favorably for clinical use [37].

### Screening pathways and assessment timing

Establishing a systematic screening pathway and identifying key assessment timepoints are essential for the effective recognition and management of cancer-related fatigue in HCC patients following TACE [35, 36]. Integrating CRF assessment into the routine perioperative nursing pathway of TACE and constructing a comprehensive management model encompassing "baseline screening at admission—regular postoperative assessment—long-term post-discharge follow-up" represents the currently recommended practice direction [37]. This model facilitates the early detection of fatigue symptoms and timely initiation of individualized supportive measures. Key assessment timepoints should span the entire treatment cycle. First, conducting a baseline assessment prior to TACE is indispensable, as it provides an important reference for subsequent evaluation of treatment-related fatigue changes. The acute phase of 24 to 72 h post-procedure is a critical window for assessing treatment-induced fatigue responses. Additionally, regular assessments at one month after the initial procedure and before each subsequent treatment cycle are equally important for monitoring the long-term trajectory of fatigue and cumulative treatment effects [37]. The screening process should organically integrate patients' subjective complaints with standardized instrument scores. For patients identified with clinically significant fatigue (e.g., BFI ≥ 4 or NRS ≥ 4), further multidimensional assessment should be conducted to identify contributing physiological, psychological, nutritional, and functional factors and to guide appropriate intervention [14, 37, 59]. Research has demonstrated a close association between fatigue and psychological distress; in symptom networks, psychological distress frequently serves as a core symptom, while somatic symptoms such as nausea may act as "bridge symptoms" that exacerbate patients' fatigue experience [38]. Therefore, the purpose of screening extends beyond quantifying the severity of fatigue to gaining a deeper understanding of the complex physiological, psychological, and social factors underlying it, thereby providing a solid foundation for developing precise, individualized non-pharmacological intervention strategies. For specific perioperative assessment timepoints and corresponding action plans, see Table 3.

## Research evidence and practice of non-pharmacological intervention strategies

### Exercise interventions

For HCC patients undergoing transarterial chemoembolization (TACE), aerobic exercise and resistance training have been most consistently supported across randomised trials and meta-analyses as effective non-pharmacological interventions for alleviating cancer-related fatigue (CRF) [39, 60, 61]. A landmark meta-analysis by Mustian et al. [61] directly compared exercise, psychological, and pharmacological interventions for CRF across 113 unique studies (*n* = 11,525) and found that exercise and psychological interventions significantly outperformed pharmacological treatment, with exercise demonstrating the largest overall effect size. The ESMO Clinical Practice Guidelines [60] similarly assign the highest recommendation grade to exercise as a first-line non-pharmacological intervention for CRF. Potential mechanisms include modulation of fatigue-related inflammatory signaling and improvements in cardiopulmonary fitness, muscle strength, and endurance, thereby countering the bidirectional fatigue-deconditioning loop. Exercise should not, however, be described as reversing muscle wasting on its own, because cancer-associated muscle loss may also be driven by anorexia, inadequate intake, tumor- and treatment-related inflammation, and catabolic metabolism [42]. In clinical nursing practice, developing individualized exercise prescriptions is essential. Exercise prescriptions must adhere to strict principles of individualization. Moderate-intensity exercise is generally recommended, with a frequency of 3–5 sessions per week and a duration of 20–45 min per session. For patients with poor hepatic functional reserve (e.g., Child–Pugh class B) or inadequate performance status, exercise programs should begin with low-intensity, short-duration activities, such as bedside exercises or Tai Chi, to ensure safety and adherence [30]. Studies have shown that exercise programs conducted under professional supervision yield superior fatigue-relieving effects compared to unsupervised programs. With the development of mobile health technologies, such as application-based reminders and wearable device monitoring, new and feasible approaches have been provided for patients to engage in scientific exercise management at home, which helps improve the continuity and effectiveness of interventions [39].

### Psychosocial interventions

Cognitive behavioral therapy (CBT) helps post-TACE patients identify and modify negative cognitions about fatigue (e.g., "fatigue means my condition is worsening") and avoidance behaviors, effectively managing the psychological distress caused by fatigue and enhancing coping abilities [40]. Mindfulness-based stress reduction (MBSR), through systematic meditation training, guides patients to enhance their awareness and non-judgmental acceptance of present-moment fatigue, thereby reducing emotional distress [39]. Health education serves as a foundational component of psychosocial interventions, enabling patients to understand that CRF is a common response during HCC treatment rather than a result of personal weakness, which helps reduce unnecessary fear and self-blame while enhancing confidence in self-management [41]. Supportive group therapy provides a platform for emotional expression, experience sharing, and social support, alleviating patients' sense of isolation [39]. These interventions can be led by oncology specialist nurses, psychotherapists, or social workers, delivered in group or individual counseling formats, and integrated into routine outpatient follow-up systems to achieve continuous attention to and support for patients' physical and psychological well-being.

### Nutritional management and traditional chinese medicine non-pharmacological therapies

For patients with HCC following TACE, nutritional management should begin with regular nutritional screening and individualized assessment to identify anorexia, nutrition-impact symptoms, inadequate dietary intake, malnutrition, and cachexia [42]. Based on the assessment findings, nurses should collaborate with physicians and clinical dietitians to provide individualized dietary counselling aimed at meeting energy and protein requirements, managing nutrition-impact symptoms, and maintaining oral intake [42]. When oral intake remains inadequate, oral nutritional supplements may be considered under professional guidance; omega-3 fatty acid-enriched formulations may be considered for selected patients, although evidence for clinically important outcomes remains limited [42]. In patients with advanced cirrhosis or protein intolerance, oral branched-chain amino acid supplementation may also be considered under specialist guidance [65]. A study involving patients with advanced HCC undergoing TACE reported that a bundled nutritional intervention was associated with improved nutritional status, reduced CRF, and better quality of life [15]. Because cachexia-related loss of skeletal muscle mass and function is driven by both inadequate intake and systemic inflammatory and metabolic abnormalities, nutritional support should be incorporated into a multimodal management strategy rather than regarded as a sufficient standalone intervention [42].

Traditional Chinese medicine-related non-pharmacological approaches have been explored as complementary strategies for CRF. However, current guideline conclusions regarding acupuncture-related and mind–body interventions remain inconsistent, and direct evidence in patients with HCC following TACE is particularly limited [60]. For example, acupoint massage (commonly targeting Zusanli and Guanyuan), auricular acupressure, moxibustion, and Tai Chi exert their effects through mechanisms that may relate to the TCM theory of harmonizing qi and blood and balancing yin and yang, as well as the modern medical evidence of regulating neuroendocrine function and reducing systemic inflammation [39]. As complementary and alternative medicine approaches, these therapies should be performed under the guidance of qualified TCM practitioners and emphasized to be integrated with conventional TACE treatment and follow-up. However, their precise efficacy and optimal implementation protocols remain to be further validated and optimized through more rigorously designed, high-quality randomized controlled trials (RCTs) Table 2.

**Table 2:** Summary of non-pharmacological interventions for CRF in Post-TACE HCC patients

Intervention Category	Specific Methods	Mechanisms of Action	Level of Evidence (OCEBM)	Guideline Recommendations
Exercise	Aerobic exercise (walking, Tai Chi), resistance training	Modulates pro-inflammatory cytokines (e.g., IL-6, TNF-α); improves cardiopulmonary function; enhances muscle strength and endurance; disrupts the fatigue–inactivity vicious cycle	Level I–II (multiple RCTs and meta-analyses)	NCCN: recommended as a primary non-pharmacological intervention for all cancer patients with CRF (Version 2.2025). ESMO: Level I evidence, Grade A recommendation (Fabi et al., Ann Oncol. 2020; 31:713–723)
Psychosocial Interventions	Cognitive behavioral therapy (CBT), mindfulness-based stress reduction (MBSR), psychoeducation, supportive group therapy	Modifies maladaptive cognitions about fatigue (e.g., “fatigue means disease progression”); enhances self-efficacy; reduces emotional distress (anxiety, depression); promotes active coping strategies	Level I–II (supported by meta-analyses)	NCCN: recommended for all patients with moderate-to-severe CRF. ESMO: Level I evidence, Grade A recommendation
Nutritional Management	Individualized dietary counselling; high-energy, high-protein foods; oral nutritional supplements when oral intake remains inadequate; omega-3 fatty acid-enriched formulations for selected patients; oral BCAA supplementation for patients with advanced cirrhosis or protein intolerance under specialist guidance	Addresses inadequate energy and protein intake and malnutrition; may help maintain body weight, skeletal muscle mass, and physical function	Level III–IV (expert consensus and small-sample studies)	NCCN: recommended to screen for and treat contributory factors including malnutrition and dehydration. ESMO: no specific grade established for nutrition alone in CRF management
Traditional Chinese Medicine (TCM) Non-Pharmacological Therapies	Acupoint massage (e.g., Zusanli, Guanyuan), auricular acupressure, moxibustion, Tai Chi	Regulates qi and blood circulation per TCM theory; modulates neuroendocrine function; attenuates systemic inflammatory responses	Level III (small-sample RCTs)	NCCN: not specifically addressed for CRF. ESMO: acupuncture recommended at Level II evidence, Grade B for CRF management

Abbreviations: *CRF* Cancer-related fatigue, *HCC* Hepatocellular carcinoma, *TACE* Transarterial chemoembolization, *CBT* Cognitive behavioral therapy, *MBSR* Mindfulness-based stress reduction, BCAA Branched-chain amino acid, *TCM* Traditional Chinese medicine, *NCCN* National Comprehensive Cancer Network, *ESMO* European Society for Medical Oncology, *RCT* Randomized controlled trial, *IL-6* Interleukin-6, *TNF-α* Tumor necrosis factor-alpha. Levels of evidence are classified according to the Oxford Centre for Evidence-Based Medicine (OCEBM) criteria: Level I (systematic reviews/meta-analyses of RCTs), Level II (individual RCTs or high-quality cohort studies), Level III (case–control, cross-sectional studies, or small-sample RCTs), Level IV (expert consensus, clinical guidelines, or case reports). NCCN recommendations are based on the NCCN Clinical Practice Guidelines in Oncology: Cancer-Related Fatigue (Version 2.2025). ESMO recommendations and grades are based on Fabi et al. (Ann Oncol. 2020;31:713–723).

## Construction of a nurse-led CRF nursing management model

### Comprehensive, individualized nursing assessment and planning

The core of constructing a nurse-led cancer-related fatigue nursing management model lies in implementing comprehensive, individualized assessment and planning Table 3. Nurses should initiate CRF risk assessment upon patient admission, establish detailed fatigue management records documenting key data including baseline fatigue levels, psychological status, and social support [43]. During the TACE perioperative period, nurses need to closely monitor the dynamic changes in patients' fatigue symptoms and identify potential precipitating factors, such as treatment-related adverse effects or psychological fluctuations. Research has demonstrated that postoperative cancer-related fatigue isgenerally prevalent among HCC patients, and its severity is closely associated with disease course, level of disease awareness, and receipt of adjuvant chemotherapy [43, 44]. Based on comprehensive assessment results, nurses should collaboratively develop individualized nursing plans with patients, their families, and the multidisciplinary team comprising physicians, dietitians, and psychotherapists. The objectives of the plan should be specific and clear, including controlling fatigue intensity, reducing fatigue interference with daily functioning, and enhancing patients' self-management abilities. For instance, the plan should specify concrete intervention measures such as appropriate exercise types and frequency, methods of psychological support, implementers, expected goals, and timepoints for reassessment. Such assessment-based, individualized planning forms the foundation for effective CRF management and improved quality of life [16]. Table 3 summarizes the key assessment timepoints and corresponding clinical actions.

**Table 3:** Key assessment timepoints and clinical actions for CRF management during the TACE perioperative period

Assessment Timepoint	Assessment Tools	Key Assessment Content	Score Threshold	Subsequent Actions
Pre-TACE (baseline)	BFI + ECOG PS + Child–Pugh	Fatigue baseline level; performance status; hepatic functional reserve	BFI ≥ 4 (moderate-to-severe fatigue)	Initiate multidimensional assessment; develop individualized intervention plan
Post-TACE 24–72 h (acute phase)	BFI/NRS	Treatment-related acute fatigue response; post-embolization syndrome symptoms	NRS ≥ 4 (moderate-to-severe fatigue)	Pain management; psychological support; early mobilization guidance; symptom monitoring
Post-TACE 1 month	BFI + EORTC QLQ-C30	Fatigue trajectory; HRQoL changes; treatment recovery status	BFI ≥ 4 (moderate-to-severe fatigue)	Adjust intervention plan; reinforce follow-up schedule; evaluate treatment response
Before subsequent treatment cycles	BFI	Cumulative treatment effect; fatigue trend across multiple TACE sessions	BFI ≥ 7 (severe fatigue)	Multidisciplinary team consultation; consider treatment regimen adjustment; intensive supportive care

Abbreviations: *CRF* Cancer-related fatigue, *TACE* Transarterial chemoembolization, *BFI* Brief Fatigue Inventory, *NRS* Numeric Rating Scale, *ECOG PS* Eastern Cooperative Oncology Group Performance Status, *EORTC QLQ-C30* European Organisation for Research and Treatment of Cancer Quality of Life Questionnaire–Core 30, *HRQoL* Health-related quality of life.Score thresholds are informed by established clinical guidelines and validation studies. For the BFI, the thresholds refer to the worst-fatigue item (0–10 scale), with scores of 4–6 indicating moderate fatigue and scores of 7–10 indicating severe fatigue (Mendoza et al., Cancer. 1999;85:1186–1196). According to the NCCN Guidelines for Cancer-Related Fatigue (Version 2.2025), NRS scores of 4–6 indicate moderate fatigue, while scores of 7–10 indicate severe fatigue. Scores of ≥4 on either measure indicate moderate-to-severe fatigue and warrant further clinical assessment. BFI ≥7 indicates severe fatigue and may warrant multidisciplinary consultation according to clinical needs. ECOG PS and Child–Pugh scores provide complementary clinical context for interpreting fatigue severity in the post-TACE population. This assessment schedule aligns with the NCCN recommendation for regular fatigue screening throughout the cancer treatment trajectory.

### Implementation and coordination of nursing interventions

**Fig. 2:**
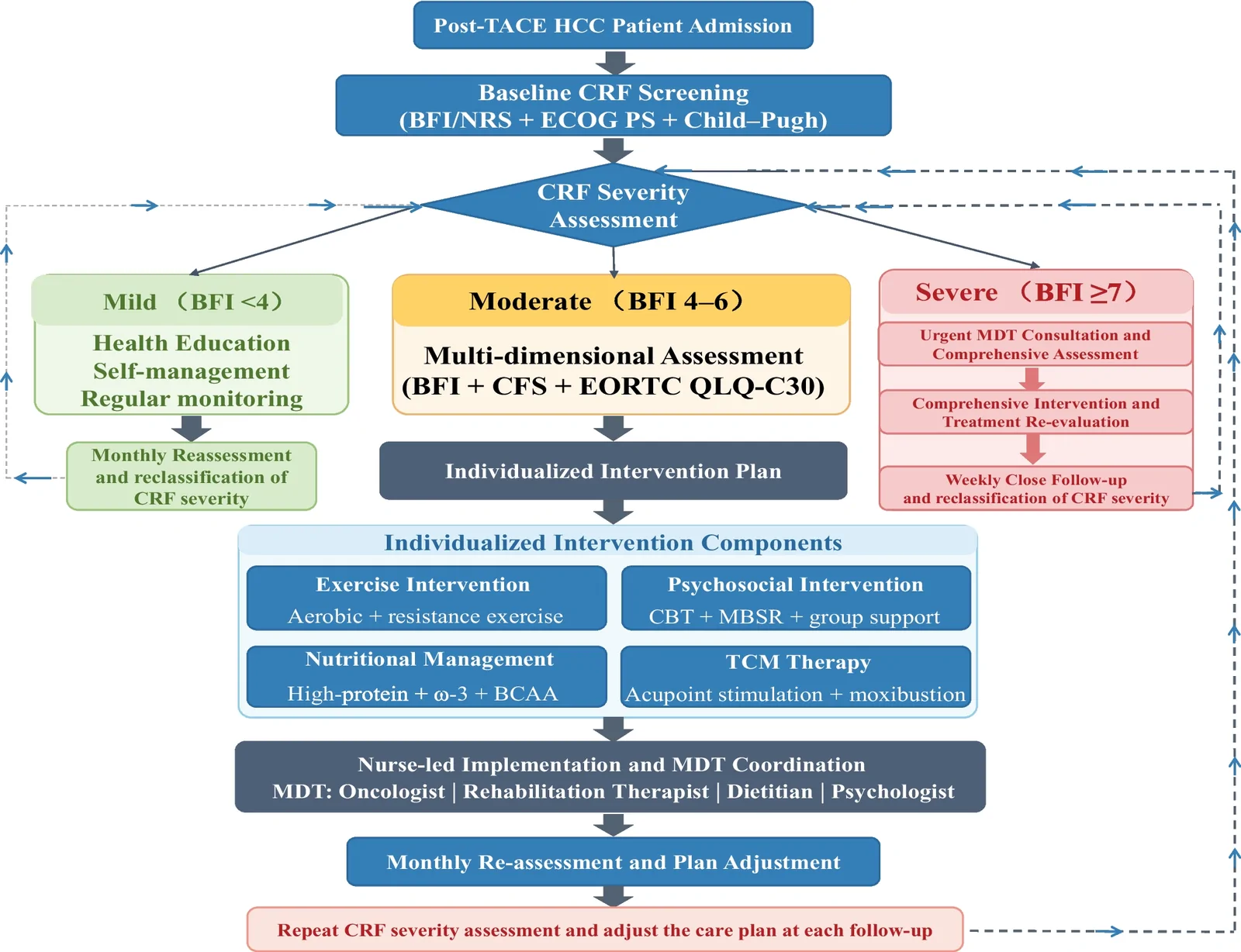
Nurse-Led Multidisciplinary CRF Management Framework for Post-TACE HCC Patients. Abbreviations: BFI, Brief Fatigue Inventory; NRS, Numeric Rating Scale; ECOG PS, Eastern Cooperative Oncology Group Performance Status; CFS, Cancer Fatigue Scale; EORTC QLQ-C30, European Organisation for Research and Treatment of Cancer Quality of Life Questionnaire; CBT, Cognitive Behavioral Therapy; MBSR, Mindfulness-Based Stress Reduction; MDT, Multidisciplinary Team; TCM, Traditional Chinese Medicine; BCAA, Branched-Chain Amino Acids

During the implementation and coordination phase of nursing interventions, nurses assume the dual role of core executor and coordinator. As the primary executor, nurses are responsible for guiding patients in safe and effective exercise training, such as aerobic exercise or walking exercise, to alleviate fatigue [16, 17]. Simultaneously, nurses should provide structured health education to help patients develop a correct understanding of their disease and treatment, and implement simple relaxation techniques such as progressive muscle relaxation to reduce anxiety and stress [19]. When patients present with severe fatigue or complicated psychological problems, the coordinating role of nurses becomes critical. At such times, nurses are responsible for promptly liaising with physicians, dietitians, psychotherapists, and other relevant professionals, ensuring that multidisciplinary collaborative interventions proceed smoothly and efficiently to jointly address patients' complex needs. Furthermore, utilizing modern communication tools such as telephone and WeChat for post-discharge follow-up is a key component of continuity of care and consolidation of therapeutic effects. Through regular follow-up, nurses can supervise patients' compliance with intervention plans, provide ongoing professional support and guidance, and timely adjust management strategies based on changes in patients' fatigue symptoms and quality of life feedback [45]. This comprehensive coordination and management spanning pre-hospital, in-hospital, and post-discharge periods can effectively enhance intervention adherence and effectiveness, ultimately helping HCC patients after TACE better cope with cancer-related fatigue and improve overall rehabilitation outcomes Fig. 2.

## Challenges, future research directions, and clinical translation

### Current challenges in clinical practice

Although transarterial chemoembolization (TACE) is an important treatment modality for hepatocellular carcinoma (HCC), the management of post-TACE cancer-related fatigue (CRF) in clinical practice still faces multiple challenges. First, inadequate attention to CRF in clinical practice is widespread, with medical resources primarily concentrated on anti-tumor treatment itself, such as optimization of TACE techniques and exploration of combination therapy regimens [22, 30]. Second, the lack of systematic CRF management pathways leads to non-standardized use of assessment tools and difficulty in sustaining intervention measures. Although some studies have focused on TACE efficacy and prognostic factors, such as the impact of tumor size, portal vein thrombosis, and hepatic functional status on survival [46], systematic assessment and interventional research targeting patients' fatigue symptoms remain scarce. Additionally, the professional resources required for non-pharmacological interventions, such as rehabilitation therapists and psychologists, have poor accessibility within the healthcare system, and patients often have low willingness for fatigue self-reporting and poor treatment adherence, making it difficult to implement effective fatigue management strategies. Limited investment in supportive care within the healthcare system means that fatigue management is often deprioritized relative to anti-tumor treatment, further restricting the implementation and effectiveness of comprehensive interventions [35].

### Future research priorities and directions

Future research should focus on constructing a more comprehensive CRF management system for HCC patients following TACE. A key research priority is to develop and validate CRF assessment tools specifically for HCC patients undergoing TACE, followed by cross-cultural validation across different populations and healthcare settings to improve the standardization and generalizability of CRF assessment [47]. Simultaneously, large-scale, multicenter randomized controlled trials should be conducted to compare the efficacy and cost-effectiveness of different non-pharmacological intervention combinations (e.g., exercise, psychological interventions, and nutritional support) to provide high-level evidence for clinical practice. Exploring potential biomarkers of CRF, such as specific cytokine profiles, to achieve objective assessment of fatigue is another key direction [48]. In terms of research methodology, leveraging artificial intelligence and machine learning technologies to predict high-risk CRF patients based on electronic medical record data will facilitate early identification and precision intervention [49]. Digital therapeutics, such as mobile-based cognitive behavioral therapy programs and virtual reality relaxation training, warrant in-depth investigation for their effectiveness in fatigue management due to their convenience and accessibility [50]. Furthermore, exploring the role of family caregivers in CRF management and their support strategies is essential for constructing a patient- and family-centered, whole-disease-course management model [51]. These research endeavors will advance supportive care for post-TACE patients and ultimately improve their quality of life and long-term prognosis.

## Conclusion

The management of cancer-related fatigue (CRF) following transarterial chemoembolization (TACE) for hepatocellular carcinoma (HCC) represents a complex challenge spanning the bio-psycho-social dimensions throughout the entire treatment course. Through comprehensive synthesis of the current literature, this review concludes that the core lies in understanding the multifactorial pathogenesis of CRF and constructing a complete closed-loop management system from precise assessment to comprehensive intervention, with the inflammatory pathway as the key hub.

First, an evidence-based standardized screening and dynamic assessment system must be established. The subjectivity and multidimensionality of CRF dictate the limitations of single indicators or one-time assessments. Therefore, clinical practice should integrate generic fatigue instruments with clinical objective indicators reflecting inflammatory status, nutritional status, and organ function, and implement regular monitoring during the pre-TACE, post-TACE, and long-term follow-up periods. This is not only the foundation for identifying high-risk patients and quantifying fatigue severity but also the prerequisite for evaluating intervention effectiveness and achieving individualized adjustment. The current challenge lies in how to seamlessly embed this assessment pathway into busy clinical workflows and enhance healthcare professionals' attention to and ability to recognize CRF.

Regarding intervention strategies, the accumulating evidence predominantly supports non-pharmacological interventions as the first-line cornerstone. Individualized exercise prescriptions, such as combined aerobic and resistance training, have been demonstrated to effectively improve physical fitness, regulate inflammatory levels, and enhance psychological status. Psychosocial interventions, including cognitive behavioral therapy and mindfulness-based stress reduction, help patients reframe their cognitions about fatigue and enhance self-management efficacy.Meanwhile, professional nutritional assessment and support are essential for addressing anorexia, malnutrition, and cachexia-related loss of skeletal muscle mass and function, while individualized exercise may counter fatigue-related reductions in physical activity and physical deconditioning. Together, these complementary strategies may help prevent further aggravation of fatigue. The advantages of these interventions lie in their high safety, good accessibility, and synergistic improvement of patients' overall health-related quality of life. However, their effective implementation is highly dependent on professional guidance and patients' long-term adherence, which precisely constitutes the main obstacle in current clinical practice.

To ensure that the above assessment and intervention measures are translated from research evidence into clinical effectiveness, an efficiently coordinated operational model must be relied upon. Constructing a multidisciplinary team-based comprehensive management model with oncology specialist nurses as the core coordinators, encompassing medical oncologists/interventional radiologists, rehabilitation therapists, clinical dietitians, psychologists/social workers, has been proposed as a promising operational framework in line with international CRF practice guidelines [62]. Nurses play an irreplaceable role in patient education, routine assessment, protocol execution, and follow-up. This model ensures that patients receive continuous, coordinated, and individualized support across different treatment stages, thereby overcoming the limitations of single-discipline perspectives and achieving resource integration and benefit maximization. It should be acknowledged, however, that direct evidence from high-quality randomised controlled trials validating a nurse-led multidisciplinary CRF management model specifically in the post-TACE HCC population remains limited; current recommendations are largely extrapolated from broader oncology supportive-care evidence and expert consensus [60, 62].

Looking ahead, research and practice in CRF management for HCC patients following TACE need to advance in greater depth. At the research level, there is an urgent need for more high-quality, large-sample randomized controlled trials, particularly interventional studies addressing TCM syndrome-based subtyping and CRF phenotypic heterogeneity in the post-TACE population, to further elucidate the biological basis and validate the efficacy of personalized protocols. At the clinical translation level, the focus should be on developing actionable clinical pathways and TACE-specific CRF assessment tools, leveraging mobile health technologies and digital therapeutics to facilitate real-time symptom monitoring and self-management, and conducting systematic training for healthcare professionals to overcome practice barriers. The ultimate goal is to promote the integration of standardization and personalization in CRF management strategies, making it an indispensable component of comprehensive TACE treatment, thereby meaningfully improving patients' long-term functional status, treatment tolerance, and overall quality of life.

## Data Availability

No datasets were generated or analysed during the current study.
